# Evaluating the Efficacy of Combined Intravaginal Estriol Therapy and Kegel Exercises in Managing Menopausal Atrophic Vulvovaginitis

**DOI:** 10.3390/clinpract15010020

**Published:** 2025-01-15

**Authors:** Lucian Șerbănescu, Vadym Rotar, Dragoș Brezeanu, Sebastian Mirea, Elena-Valentina Ionescu, Paris Ionescu

**Affiliations:** 1 Faculty of Medicine, Ovidius University of Constanta, 900470 Constanta, Romania; rotarvadym.obst.gyn@gmail.com (V.R.); brezeanudragos@gmail.com (D.B.); elena.ionescu@365.univ-ovidius.ro (E.-V.I.); paris.ionescu@365.univ-ovidius.ro (P.I.); 2 County Clinical Emergency Hospital “Sf. Ap. Andrei”, 900591 Constanta, Romania; sebastian.mirea@umft.ro; 3 Department of Rehabilitation, Balneal and Rehabilitation Sanatorium of Techirghiol, 906100 Techirghiol, Romania

**Keywords:** atrophic vulvovaginitis, estriol therapy, Kegel exercises, menopause, vulvovaginal atrophy, postmenopausal symptoms, estrogen deficiency, pelvic floor exercises

## Abstract

**Background**: This is a prospective study. Atrophic vulvovaginitis (VVA), a prevalent condition resulting from estrogen deficiency after the menopause, is characterized by symptoms such as vaginal dryness, itching, burning, dyspareunia, and urinary discomfort. Standard treatment involves systemic estrogen replacement therapy (HRT) and localized estrogen treatments, such as estriol. However, many women with moderate-to-severe VVA may not fully benefit from estrogen therapy alone. Non-hormonal adjunctive treatments, such as pelvic floor exercises (e.g., Kegel exercises), are being explored to enhance clinical outcomes. **Objectives**: This study investigates the combined effect of local estriol therapy and Kegel exercises in improving VVA symptoms in postmenopausal women. **Methods**: Fifty postmenopausal women diagnosed with VVA were enrolled and divided into three severity groups: mild, moderate, and severe. All participants received estriol therapy (0.5 mg vaginal tablets daily for 10 days each month) for the first three months. Following this, Kegel exercises were introduced for an additional three-month period, alongside continued estriol therapy. Symptom improvement was evaluated after six months, with outcomes categorized as complete remission, partial remission, or no remission. **Results**: Significant improvements in symptom remission were observed, particularly in the moderate and severe groups. In the mild VVA group, 81.82% achieved complete remission with combined therapy compared to 68.18% with estriol alone. In the severe group, complete remission was observed in 40% of patients receiving combined therapy compared to 20% with estriol therapy alone. These findings suggest that Kegel exercises enhance the effectiveness of estriol by improving local blood circulation, which facilitates better estrogen absorption and distribution. **Conclusions**: The addition of Kegel exercises to local estriol therapy significantly improves symptom remission rates, especially in moderate and severe VVA cases. This approach offers a promising strategy for managing postmenopausal VVA, particularly in cases that do not fully respond to estrogen therapy alone.

## 1. Introduction

Atrophic vulvovaginitis, or vulvovaginal atrophy (VVA), is a common manifestation of the genitourinary syndrome of menopause (GSM), a condition resulting from estrogen deficiency following the menopause [1,2]. It is characterized by a spectrum of symptoms including vaginal dryness, itching, burning, dyspareunia (painful intercourse), and urinary symptoms [1]. These symptoms are due to the degenerative changes occurring in the vulvar and vaginal tissues, which are primarily mediated by a reduction in estrogen levels [2,3]. The decline in estrogen leads to a decrease in the thickness and elasticity of the vaginal epithelium, reduced glandular secretions, and impaired vascular supply, culminating in vaginal atrophy [2,4].

Estrogen plays a pivotal role in maintaining the integrity of the vaginal mucosa and its associated structures. Estrogen receptors (ERα and ERβ) are abundantly expressed in the urogenital tissues, particularly in the vaginal epithelium and the smooth muscle fibers of the pelvic floor, making the vaginal and vulvar tissues highly sensitive to estrogenic signals. In the absence of estrogen, the vaginal epithelium undergoes thinning, leading to a decrease in glycogen storage, which is essential for maintaining a low vaginal pH (5–5.5), a condition which supports lactobacilli growth [4,5]. The reduction in lactobacilli results in an increase in vaginal pH and a predisposition to infections. Furthermore, diminished estrogen levels impair collagen production and alter the composition of extracellular matrix proteins, leading to decreased vaginal elasticity and potential for prolapse in more severe cases [4].

Systemic estrogen replacement therapy (HRT) and localized estrogen treatments, such as intravaginal estriol, are important tools of management for VVA. Estriol, a weak estrogen, is often used for its high local efficacy and minimal systemic absorption, making it suitable for long-term therapy without the risks associated with systemic estrogen administration [4]. Estriol has been shown to restore mucosal integrity, enhance vaginal blood flow, improve vaginal pH, and reduce the clinical symptoms associated with vaginal atrophy. Despite its benefits, many women with VVA present with moderate-to-severe symptoms that may not be fully alleviated by estrogen therapy alone [5,6,7].

In this context, non-hormonal adjunctive therapies, such as pelvic floor exercises (e.g., Kegel exercises), have gained attention for their potential to enhance the clinical outcomes of local estrogen treatment. Kegel exercises, which involve voluntary contraction and relaxation of the pelvic floor muscles, aim to improve muscle tone, increase local blood flow, and enhance tissue trophism [8,9]. The pelvic floor muscles play a crucial role in supporting the urogenital organs and promoting vascular circulation in the vaginal area [10,11]. Studies suggest that Kegel exercises may enhance the efficacy of local estrogen therapy by improving blood flow, which could facilitate better estrogen absorption and distribution, particularly in women with severe or moderate symptoms where both hormonal deficiency and insufficient blood supply are contributing factors to the condition [3,12,13,14].

Combining estriol therapy with Kegel exercises may provide a comprehensive approach to managing VVA, addressing both the hormonal and physical aspects of tissue atrophy [15,16]. This combination therapy could potentially lead to greater improvements in symptom relief, particularly in women with moderate-to-severe VVA where estrogen alone may not sufficiently restore tissue health. Given the multifactorial nature of VVA, a combined therapeutic strategy may optimize outcomes by improving both local estrogenic effects and pelvic muscle function [6,7,17,18].

## 2. Materials and Methods

This prospective study included 50 postmenopausal women presenting with vulvovaginal symptoms at the Santerra Medical Center and the Constanța County Emergency Hospital. The study was approved by Santerra Medical Center Ethical Committee (approval no. 003, 17 January 2024) and complied with the revised ethical guidelines of the Declaration of Helsinki. All patients entered into the study agreed to participate and signed an informed consent form after all of the risks and benefits of the therapy were explained to them. They had the opportunity to stop participating at any time and their data were confidential.

The main symptoms reported were vaginal burning, itching, dyspareunia, and excessive vaginal dryness. The patients were divided into three groups based on the severity of their condition, clinical criteria during inspection, examination with valves or speculum or vaginal cough, and on the basis of a form in which the patients ticked one of the 3 headings: severe, moderate, or mild symptoms (vulvar and vaginal dryness, vaginal burning, itching, or dyspareunia). The final classification in one of the degrees of severity was made by a team of experienced senior gynecologists, which were based on both the clinical aspect and the severity of the symptoms reported by the patient.

- **Severe atrophic vaginitis**—severe vaginal stenosis, friable vulvar, or vaginal mucosa (vulvar and vaginal dryness, vaginal burning, itching, or dyspareunia);
- **Moderate atrophic vaginitis—**moderate vaginal stenosis, thin pale vulvar, or vaginal mucosa (vulvar and vaginal dryness, vaginal burning, itching, or dyspareunia);
- **Mild atrophic vaginitis—**mild or absence of vaginal stenosis, pale vulvar, or vaginal mucosa (vulvar and vaginal dryness, vaginal burning, itching, or dyspareunia).

### 2.1. Inclusion and Exclusion Criteria

- **Inclusion criteria:** Postmenopausal women with vulvovaginal symptoms.
- **Exclusion criteria:** Active urinary or genital infections, systemic health issues, recent pelvic surgeries, or recent hormone treatment.

### 2.2. Treatment Protocol

The study was conducted in two phases:

**Phase 1 (First 3 months):** Patients received local estriol therapy. Each patient was given vaginal tablets containing 0.5 mg of estriol daily for 10 days each month. This treatment was continued for a total of 3 months.

**Phase 2 (Next 3 months):** After the first 3 months of estriol treatment, patients were instructed to begin Kegel exercises in addition to continuing estriol therapy. For the first month of this phase, the Kegel exercises involved contracting the pelvic muscles for 3 s, followed by a 3 s relaxation period. Patients performed these exercises in the supine position with their thighs flexed at a 90-degree angle to the abdomen. Each session lasted 15 min and was performed twice daily. In the second and third months, both contraction and relaxation periods were extended to 5 s.

### 2.3. Follow-Up and Evaluation

After 6 months (3 months of estriol therapy followed by 3 months of combined estriol and Kegel exercises), patients were evaluated for clinical improvement, which was categorized as:

- **Complete remission;**
- **Partial remission;**
- **No remission.**

### 2.4. Data Analysis

The statistical analysis was performed using IBM SPSS version 23. Data are presented as the mean ± standard deviation (SD) for continuous variables in cases of symmetric distributions. The normality of the continuous data was estimated with Shapiro–Wilk Tests of Normality. For hypotheses testing, Independent Samples Median test, *t*-Student Test and Chi-Square Test of association were used depending on the type of analyzed variables. The significance level α was set at 0.05. If the test statistics for every test conducted was in the critical region and the *p*-value was less than or equal to the significance level, we decided to reject the null hypothesis in favor of the alternative hypothesis. In the Section 4, we present the *p*-statistic and the confidence interval only where they had an important statistical significance in terms of our observations and future conclusions. Although the number of cases studied was not very large, the accuracy of the study was significant.

## 3. Results

### 3.1. Patient Distribution by Severity of Condition

The 50 patients were divided into three groups based on the severity of their condition. Below is the distribution of patients according to the severity of atrophic vaginitis (Table 1):

### 3.2. Symptom Remission by Treatment Group

After 3 months of estriol therapy followed by Kegel exercises, the results were assessed based on clinical improvement. The remission rates by severity group are shown below (Table 2):

### 3.3. Age and Menopause Onset

The patients were also categorized based on the time elapsed since the onset of the menopause and their age (Table 3 and Table 4):

The combination of local estriol therapy and Kegel exercises resulted in improvement in vulvovaginal symptoms, especially in the moderate and severe cases. Estriol is known to improve the trophic properties of the vaginal mucosa, restoring elasticity and reducing dryness. Kegel exercises can increase the absorption of estriol due to improved local blood flow and muscle tone, which can also contribute to the better distribution of estrogen locally.

## 4. Discussion

Atrophic vaginitis (AV) is a prevalent condition in postmenopausal women, characterized by thinning, dryness, and inflammation of the vaginal walls due to reduced estrogen levels. It significantly impacts quality of life, causing symptoms such as vaginal dryness, burning, itching, dyspareunia, and urinary discomfort (urinary stress incontinence and dysuria) [1,2,6]. This condition is a consequence of the loss of estrogenic stimulation to the vaginal epithelium, leading to decreased glycogen stores and disruption of the normal vaginal microbiota [4,5,7]. The management of AV is critical to improving the symptoms and overall well-being of postmenopausal women, and this study highlights the therapeutic benefits of combining local estrogen therapy (estriol) with pelvic floor exercises (Kegel exercises) to achieve optimal clinical outcomes [10,11,12,15].

### 4.1. The Role of Estriol in the Treatment of Atrophic Vaginitis

Estrogen replacement therapy (ERT), particularly through local treatments like estriol, is widely recognized as the first-line treatment for atrophic vaginitis. Estriol is a naturally occurring estrogen that has a high affinity for estrogen receptors in the vaginal mucosa but is less likely to cause systemic side effects compared to other forms of estrogen [18,19]. Local estriol therapy helps restore vaginal epithelial integrity, improve vaginal lubrication, and increase the thickness of the vaginal walls by stimulating the production of glycogen and Lactobacillus, which enhance vaginal health [18,19,20].

The results of our study support the effectiveness of estriol in reducing symptoms of AV, particularly in mild-to-moderate cases, with a significant proportion of patients achieving complete or partial symptom remission after 3 months of treatment. These findings align with the existing literature, where studies have shown that vaginal estriol administration significantly alleviates symptoms like dryness, itching, and discomfort during intercourse [18,21]. However, despite the efficacy of estriol therapy, its effectiveness in severe cases of AV, where the vaginal epithelium is more atrophic and the blood supply is compromised, might not be as pronounced. This suggests that additional treatment modalities may be needed to optimize outcomes in these cases.

### 4.2. Kegel Exercises and Their Synergistic Role in Managing AV

Kegel exercises, known for their role in strengthening the pelvic floor muscles, have recently gained attention as an adjunctive therapy for managing atrophic vaginitis and other pelvic floor disorders. The principle behind Kegel exercises is that by strengthening the pelvic muscles, there is an improvement in local blood circulation, which enhances the delivery of oxygen and nutrients to the vaginal tissues. This improved circulation is particularly important for women with AV, as it can help counteract the insufficient blood flow that exacerbates the condition [22].

Our study demonstrates that the combination of local estriol therapy and Kegel exercises resulted in a higher rate of complete remission of symptoms, particularly in moderate and severe AV cases. The findings are consistent with previous studies, which showed that pelvic floor muscle training, including Kegel exercises, significantly improved vaginal atrophy and increased sexual satisfaction in postmenopausal women when combined with hormone therapy [23]. The proposed mechanism is that Kegel exercises enhance the transcutaneous absorption of estrogen, thereby increasing the effectiveness of the local estrogen therapy [24].

Additionally, Kegel exercises have been shown to increase muscle tone, which may help in counteracting the mechanical factors that contribute to vaginal atrophy, such as pelvic organ prolapse or decreased vaginal support. Furthermore, Kegel exercises may help reduce urinary symptoms that often coexist with atrophic vaginitis, including incontinence and urgency, which could further enhance the overall quality of life for these women [25].

### 4.3. Impact of Age and Time Since the Menopause

The results of this study also highlight the impact of age and the duration of the menopause on the severity of symptoms and the response to treatment. In the severe group, 80% of patients had been in the menopause for more than 10 years (Table 5). Our findings show that women who had experienced the menopause for a longer period (more than 10 years) had a poorer response to estrogen therapy, particularly in the severe AV group (Table 2). This is in line with other studies that suggest that prolonged estrogen deficiency leads to more significant vaginal atrophy and less responsiveness to treatment. In these women, the vaginal epithelium may become more resistant to hormonal stimulation, necessitating longer or more intensive treatments.

Interestingly, women who were within the first 5 years of the menopause showed better outcomes, particularly in the mild and moderate AV groups. This could be due to the fact that the vaginal tissues in these women are less severely affected by estrogen loss, allowing for quicker and more pronounced responses to estrogen therapy and pelvic floor exercises.

### 4.4. Clinical Implications and Future Research

The combination of local estriol therapy and Kegel exercises offers a promising approach for managing atrophic vaginitis, particularly in women with moderate-to-severe symptoms. Clinicians should consider this combined approach, especially in patients who exhibit insufficient response to estriol alone. The addition of Kegel exercises addresses the vascular insufficiency that often accompanies severe AV and helps enhance the therapeutic effects of estrogen by improving local blood flow.

However, there are some limitations to our study, including the small sample size. Further research with larger cohorts and long-term follow-up would be beneficial to confirm these findings and better understand the long-term efficacy and safety of this combined approach. Additionally, future studies could explore the potential synergistic effects of Kegel exercises with other forms of local estrogen therapy, such as estradiol or conjugated estrogens, to identify the most effective treatment regimens for different patient populations.

Also, patients who have associated gynecological pathology, ovarian or adnexal tumor formations, due to painful phenomena may be limited in performing Kegel exercises [26].

Another particularly important factor in the success of the therapy is the personality type of each patient. In general, melancholic patients or those prone to developing depression have a greater tendency to abandon therapy or not perform Kegel exercises correctly [27].

After 6 months of treatment (3 months of estriol followed by 3 months of combined estriol and Kegel exercises), the symptom remission rates were higher in the moderate and severe groups compared to the mild VVA group. In the mild VVA group, 81.82% of patients achieved complete remission after combined treatment compared to 68.18% after estriol alone. In the moderate VVA group, 55.56% of patients experienced complete remission after the combined approach compared to 38.89% with estriol alone (*p* < 0.05, CI 95%). For the severe VVA group, combined therapy resulted in a 40% complete remission rate, a significant improvement from the 20% seen with estriol therapy alone (*p* < 0.05, CI 95%). These findings demonstrate that the addition of Kegel exercises can improve the efficacy of estriol, probably by enhancing local blood flow, which may facilitate better estrogen absorption and distribution, ultimately leading to greater symptom relief in more severe cases of VVA.

## 5. Conclusions

The results of this study indicate that combining local estriol therapy with Kegel exercises can enhance the treatment outcomes for vulvovaginal atrophy (VVA), particularly in moderate and severe cases.

## Figures and Tables

**Table 1:** Distribution of patients by degree of severity of atrophic vulvar vaginitis.

Severity Group	Number of Patients
Mild	22
Moderate	18
Severe	10

**Table 2:** Distribution of patient groups according to remission of symptoms.

Severity Group	Post-Estriol Treatment	Post-Estriol + Kegel Exercises
Mild (22 patients)	15 complete, 4 partial, 3 none	18 complete, 3 partial, 1 none
Moderate (18 patients)	7 complete, 6 partial, 5 none	10 complete, 6 partial, 2 none
Severe (10 patients)	2 complete, 2 partial, 6 none	4 complete, 4 partial, 2 none

**Table 3:** Distribution of patients by age group.

Age Group	Number of Patients
45–50 years	17
50–55 years	13
55–60 years	15
60–65 years	5

**Table 4:** Distribution of patients by time elapsed since menopause onset.

Time Since Menopause Onset	Number of Patients
<5 years	15
5–10 years	18
10–15 years	12
>15 years	5

**Table 5:** Distribution of patients in the severity groups according to the period elapsed since the onset of the menopause.

Severity Group	Time Since Menopause Onset			
	<5 Years	5–10 Years	10–15 Years	>15 Years
Mild (22 patients)	14	6	2	0
Moderate (18 patients)	1	10	7	0
Severe (10 patients)	0	2	3	5

## Data Availability

The data supporting this study’s findings are available from the corresponding author, Lucian Șerbănescu, upon request.
